# Induction Heating of Magnetically Susceptible Nanoparticles for Enhanced Hydrogenation of Oleic Acid

**DOI:** 10.1021/acsanm.1c04351

**Published:** 2022-02-17

**Authors:** Cameron L. Roman, Natalia da Silva Moura, Scott Wicker, Kerry M. Dooley, James A. Dorman

**Affiliations:** † Cain Department of Chemical Engineering, Louisiana State University, Baton Rouge, Louisiana 70803, United States; ‡ Department of Chemistry, Rhodes College, Memphis, Tennessee 38112, United States

**Keywords:** induction heating, fatty acid hydrogenation, magnetic catalysis, magnetic nanoparticles, oleic acid, selectivity, coke

## Abstract

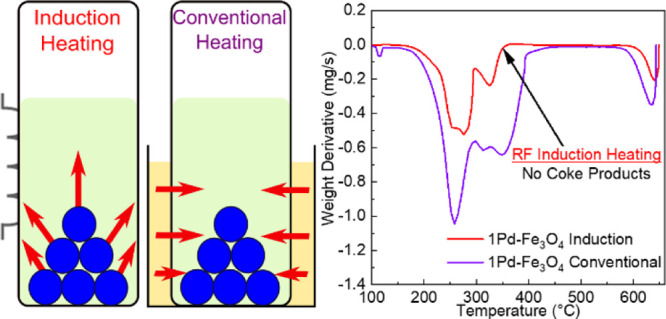

Radio frequency (RF) induction heating was compared to conventional thermal heating for the hydrogenation of oleic acid to stearic acid. The RF reaction demonstrated decreased coke accumulation and increased product selectivity at comparable temperatures over mesoporous Fe_3_O_4_ catalysts composed of 28–32 nm diameter nanoparticles. The Fe_3_O_4_ supports were decorated with Pd and Pt active sites and served as the local heat generators when subjected to an alternating magnetic field. For hydrogenation over Pd/Fe_3_O_4_, both heating methods gave similar liquid product selectivities, but thermogravimetric analysis–differential scanning calorimetry measurements showed no coke accumulation for the RF-heated catalyst *versus* 6.5 wt % for the conventionally heated catalyst. A different trend emerged when hydrogenation over Pt/Fe_3_O_4_ was performed. Compared to conventional heating, the RF increased the selectivity to stearic acid by an additional 15%. Based on these results, RF heating acting upon a magnetically susceptible nanoparticle catalyst would also be expected to positively impact systems with high coking rates, for example, nonoxidative dehydrogenations.

## 1 Introduction

Industrial processing of the longer chain fatty acids, such as lauric, palmitic, and oleic acid, traditionally focuses on decreasing the degree of unsaturation while maintaining the carboxylic acid group and avoiding isomerization.^1−3^ Industrial practice favors nickel catalysts operating between 140 and 190 °C and 2–3 bar.^1,3^ There is also interest in unsaturated alcohols as they are high-value products in pharmaceuticals, cosmetics, and household applications.^4−6^ Commercial processes using Cu–Cr-based catalysts have shown to offer good selectivity to unsaturated alcohols and high stability.^4^ Unfortunately, along with the environmental risks of Cr, high temperatures (250–350 °C) and pressures (100–200 bar) are needed to drive the process toward alcohols.^4^ Hydrogenation of the carboxylic groups (decarboxylation) is possible, producing alkanes and cracked (lower molecular weight) alkanes,^7−9^ when using transition metals such as Pd, Pt, and Ni on metal oxide or carbon supports. Typical temperatures and pressures for this application are 300–360 °C and 15–27 bar (0–5% H_2_), respectively. These catalysts are preferred when diesel-fuel-like products are desired.^7−9^

Due to the high interest in the hydrogenation of fatty acids, there are numerous papers focused on supported Pt, Pd, Rh, and Ru.^1^ While these metals are generally selective for C=C hydrogenation, minimizing defect sites and adding an electropositive promoter such as Co, Ge, Fe, Ga, Ni, or Sn both increase the metals’ electronegativity and decrease their ensemble size, increasing the selectivity to unsaturated alcohols as a result.^5,6^ Others have explored various supports such as SiO_2_, Al_2_O_3_, and metal oxides such as TiO_2_, ZnO, and CeO_2_ that can also increase selectivity to the unsaturated alcohol by either a strong metal–support interaction or the oxygen-exchange ability of the support with reactants.^6,10,11^ Both approaches have been shown to have significant effects on selectivity.^5,6,11^

Much fatty acid hydrogenation research explores the effects of temperature, pressure, and catalyst composition while still relying on thermal conduction and convection to supply the necessary energy to drive the reaction. While these methods of energy transfer are well understood in the reactor design, they are also inefficient and slow compared to nonionizing radiation.^12,13^ Radio frequency (RF) induction heating allows increased efficiency due to the high penetration capability of RF waves and their ability to induce magnetic dipoles in a magnetic material.^14,15^ As a result, RF-heated catalysts can be 90% energy-efficient in an industrial environment compared to 50% for a conventional reactor.^16^ One approach uses sub-millimeter metallic beads to act as a RF absorber which then heats the process stream and catalyst bed.^17^ Another approach is to incorporate ferrimagnetic or ferromagnetic nanoparticles into the catalyst itself and utilize the material’s magnetic hysteresis. By the latter method, the RF field localizes heat generation at the catalyst surface through hysteresis losses and minimizes the temperature gradient between the catalyst and substrate interface, which is expected to increase the overall rate of reaction.^14,15,17,18^ Furthermore, the increased temperature uniformity due to the alternating magnetic field and the Curie temperature (*T*_c_) of the material can prevent hot-spot generation leading to unwanted side reactions.^13,17,19^ While improving overall heat transfer, the constraints imposed by the magnetic material increase the difficulty of designing an effective catalyst. For example, the active metal or metal oxide crystallite size and morphology can influence hysteresis losses and their control in optimal catalyst design can conflict with the demands of induction heating.^14,15,18^ Currently, there are no known industrial applications of catalytic induction heating, but smaller-scale experiments have been published.^20,21^ For example, in methane steam reforming, induction-heated catalysis could achieve 90% methane conversion over a single catalyst at temperatures of 700–800 °C.^21^ A similar approach has been attempted with microwave heating in dry reforming catalysis, where a reduction of coke formation has been observed.^22^

This project aims to evaluate the effects magnetic nanoparticle RF induction heating has on the activity and selectivity in the hydrogenation of a typical fatty acid, oleic acid. The catalytic performances of RF and conventional thermal heating are compared for Pd and Pt supported on the microspheres of ferrimagnetic Fe_3_O_4_ nanoparticles. The Fe_3_O_4_ support, composed of ∼21 nm nanoparticles, demonstrated the expected hysteresis heating based on specific loss power (SLP) measurements, while the two noble metals have distinct selectivity characteristics for oleic acid hydrogenation.^23,24^ The catalysts were characterized by thermogravimetric analysis (TGA)–differential scanning calorimetry (DSC), chemisorption, X-ray diffraction (XRD), and X-ray photoelectron spectroscopy (XPS).

## 2 Experimental Section

### 2.1 Materials

Citric acid (C_6_H_8_O_7_, Fisher Chemicals, 99.7%) and sodium bicarbonate (NaHCO_3_, Fisher Chemicals, 100.3%) were used for the synthesis of the trisodium citrate dehydrate (C_6_H_9_Na_3_O_9_). Ferric chloride hexahydrate (FeCl_3_·6H_2_O, Fisher Chemicals, 99.7%), sodium acetate (CH_3_COONa, Curtin, >99%), and ethylene glycol (HOCH_2_CH_2_OH, Fisher Chemicals, certified) were used for the Fe_3_O_4_ solvothermal synthesis. Palladium(II)chloride (PdCl_2_, Pressure Chemical) and hydrochloric acid (HCl, Acros Organics, 37%) were used for the Pd deposition, and hydrogen hexachloroplatinate(IV)hydrate (H_2_PtCl_6_·*x*H_2_O) was used for the Pt impregnation. Hydrochloric acid (HCl, 36.5–38%, VWR Chemicals) and nitric acid (HNO_3_, 68–70%, VWR Chemicals) were used for the inductively coupled plasma optical emission spectrometry (ICP-OES) digestion process. Dodecane (C_12_H_26_, Acros Organics, >99%) and oleic acid (C_18_H_34_O_2_, Alfa Aesar, 90%) were used as the reactor solvent and reactant, respectively.

### 2.2 Preparation of 1 wt % Pd/Fe_3_O_4_ and 3 wt % Pt/Fe_3_O_4_ Catalysts

**Figure 1:**
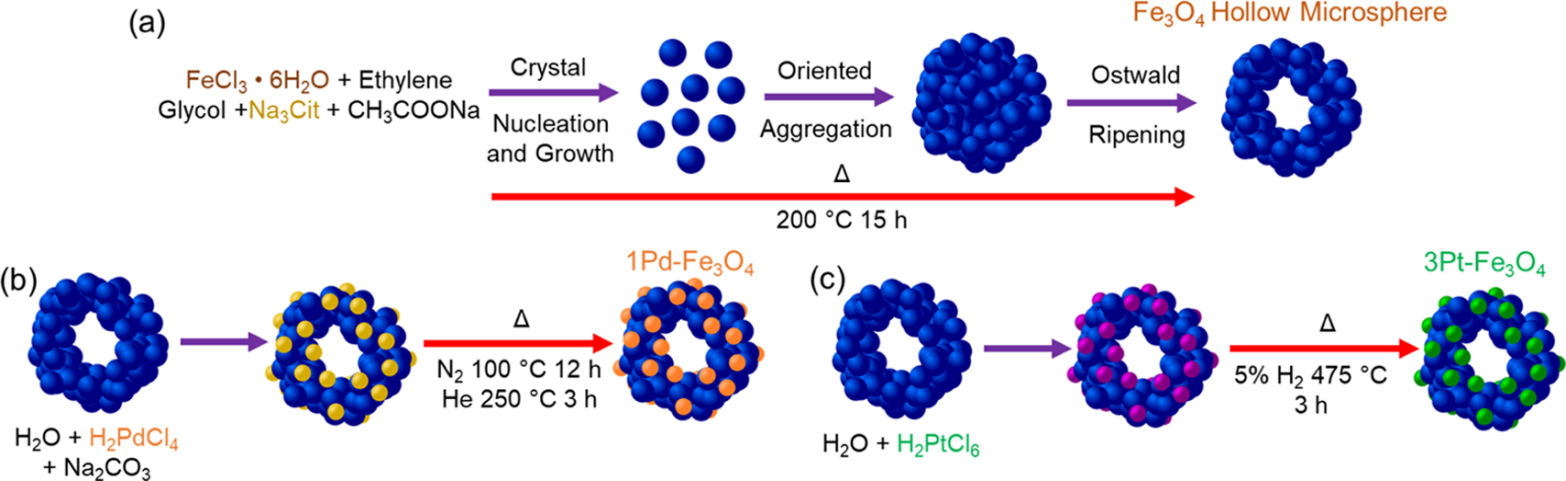
(a) Schematic illustration of the solvothermal synthesis of mesoporous Fe_3_O_4_ microspheres, (b) illustration of the Pd deposition (1Pd–Fe_3_O_4_), and (c) illustration of the Pt impregnation (3Pt–Fe_3_O_4_).

The Fe_3_O_4_ magnetic catalyst supports were synthesized following a previously reported method (Figure 1a).^25^ Specifically, 1.0 g of citric acid was dissolved in 2 mL of DI water and 1.3 g of NaHCO_3_ was dissolved in 20 mL of DI water. The two solutions were mixed, heated to 125 °C, and allowed to evaporate completely to form C_6_H_9_Na_3_O_9_. Next, 13.5 g of FeCl_3_·6H_2_O, 0.60 g of C_6_H_9_Na_3_O_9_, and 36 g of CH_3_COONa were fully dissolved in 400 mL of ethylene glycol. The mixture was transferred to a PTFE-sleeved autoclave, sealed, purged with N_2_, brought to 200 °C, and maintained for 15 h. The black precipitate was washed with DI water four times, twice with a 50:50 mixture of DI water and isopropanol, and then contacted at 80 °C with a 50:50 mixture of DI water and ethanol under N_2_ for 2 h twice. The precipitate was vacuum-dried at 60 °C for 6 h and calcined at 325 °C for 3 h in flowing N_2_.

The Pd deposition method was adapted from Freund et al. (Figure 1b).^26^ Here, a 15 mM solution of tetrachloropalladic acid (H_2_PdCl_4_) was created by dissolving 1.0 g of PdCl_2_ in a 70:100 mixture of 37 wt % HCl and DI water. The solution was then diluted to 15 mM with DI water, and 1.28 g of iron oxide support was suspended in 8.1 mL of this solution for 6 h to give a nominal 1.0 wt % Pd catalyst (1Pd–Fe_3_O_4_). The pH was brought to 10 with 1 M sodium carbonate, and then the solution was heated to 70 °C overnight to reduce the volume by 15–25%. The precipitate was then washed four times with DI water, centrifuged, and heated at 100 °C in flowing N_2_ for 12 h. The powder was then reduced at 250 °C in flowing He for 3 h.

For the Pt impregnation, 98.5 mg of H_2_PtCl_6_·6H_2_O was dissolved in 60 mL of DI water to give nominal 3 wt % Pt (3Pt–Fe_3_O_4_) for 1.20 g of iron oxide support (Figure 1c). The solution was added dropwise to the support and allowed to slowly evaporate at 70 °C. The catalyst was then dried for 12 h at 100 °C and then reduced at 475 °C in 5% H_2_/N_2_ for 3 h.

### 2.3 Structural Characterization

The Fe_3_O_4_ content was estimated by TGA–DSC (TA SDT Q600) using temperature-programmed oxidation (TPO). In flowing N_2_, the sample was equilibrated at 250 °C, ramped at 10 °C min^–1^ to 325 °C, held for 60 min, and then ramped at 10 °C min^–1^ to 400 °C, with a 30 min final hold. This treatment prior to oxidation was designed to remove any residual water and organics that may remain after synthesis. The gas was then switched to air for 60 min and then ramped to 600 °C at 10 °C min^–1^.

Surface area and pore volume measurements were performed on a Micrometrics ASAP 2020 utilizing the Brunauer–Emmett–Teller method. Pd and Pt dispersion was quantified by pulse chemisorption on a Micromeritics Pulse Chemisorb 2700, Pd with CO (assumed 1 CO/Pd) and Pt with H_2_ (assumed 1 H/Pt). The morphology and size of the Fe_3_O_4_ microspheres were measured by high-resolution transmission electron microscopy (HRTEM) using a JEOL JEM-1400 operating at 120 kV and using an Orius Camera SC1000A 1, with a 0.20 nm lattice image resolution and a 0.38 nm point image resolution. The powder sample was dispersed in ethanol and drop-cast on a 300 mesh, lacey carbon grid prior to imaging. The elemental composition of the catalysts was measured using a Perkin Elmer Optima 8000 inductively coupled plasma optical emission spectrometer equipped with an autosampler. Samples for ICP-OES analyses were prepared by digesting 2.1 mg of 1 wt % Pd/Fe_3_O_4_ and 30.0 mg of 3Pt–Fe_3_O_4_ in a HNO_3_ (68–70%) and HCl (36.5–38%) solution, which is heated to ∼70 °C. 1Pd–Fe_3_O_4_ is diluted to 45 ppm Fe and 0.63 ppm Pd, respectively, and 3Pt–Fe_3_O_4_ is diluted to 16 ppm for both Fe and Pt using 2% HNO_3_. Powder XRD data were obtained using the Cu Kα1 (λ = 1.54 Å) emission on a PANalytical XRD with a step size of 0.04° and a dwell time of 60 s. XPS results were collected using a Scienta Omicron ESCA 2SR XPS equipped with a monochromatic Al Kα (*h*ν = 1486.6 eV) X-ray source and a hemispherical analyzer with a 128-channel detector. The inherent Gaussian width of the photon source was 0.2 eV. The pressure inside the chamber was 1.5 × 10^–9^ Torr. The XPS spectra were calibrated to the adventitious C 1s peak at 284.8 eV, and peak deconvolution was performed (using the CasaXPS software) using Gaussians after Shirley background subtraction.

### 2.4 Catalytic Performance Evaluation

Batch reactions were performed with 500 mg of oleic acid, 5.00 g of dodecane solvent, and 50.0 mg of the catalyst in a glass vial. The vial was purged with N_2_ for 30 min and then H_2_ at 1 atm for 30 min before heating. The induction heating was driven using an Ambrell EASYHEAT 8130LI 10 kW induction heater (0–600 A, 343 kHz) with a 3-turn, 0.035 m diameter Cu coil. The bulk temperature was measured using a Neoptix T1 PTFE-coated fiber optic cable. The current was adjusted based on the desired bulk temperature (70, 110, and 150 °C) for 4 h. A conventional thermal reaction (oil bath) was used as control with identical temperatures and times.

For chromatographic analysis, each sample was derivatized with *N*,*O*-bis(trimethylsilyl)acetamide (BSA) at a molar ratio of 4:1 BSA to oleic acid. Pyridine was added, at 1/10th the volume of BSA, to catalyze the derivatization, which created a nonpolar trimethylsilyl ester (from the acids and aldehydes) or ether (from the alcohols). Product identification was performed by mass spectrometry using an Agilent GC 6890N/Agilent MSD 5975B. A Wasson KC066 (0.25 mm diameter, 100 m long) dimethylsiloxane column was used at a 15:1 split ratio at 13.3 mL min^–1^ total flow. The temperature was ramped at 15.0 °C min^–1^ from 100 to 230 °C with a 92 min hold time. Product quantification was performed on an HP 5890 Series II GC with an FID and an HP 7673 autosampler fitted with a Supelco Equity-1 (0.32 mm diameter, 30 m long) column. A split ratio of 50:1 was used at a total flow of 64.8 mL min^–1^. The temperature was ramped at 7.0 °C min^–1^ from 100 to 230 °C with a hold time of 11.5 min. TPO (TA SDT Q600) was used to quantify the coke and coke-like products on the catalyst surface. Before running the TPO, the catalysts were vacuum-dried at 110 °C for 12 h. The sample was equilibrated and held for 30 min at 100 °C in N_2_, switched to air, ramped at 10 °C min^–1^ to 650 °C, and held for 50 min.

## 3 Results and Discussion

### 3.1 Catalyst Characterization

**Figure 2:**
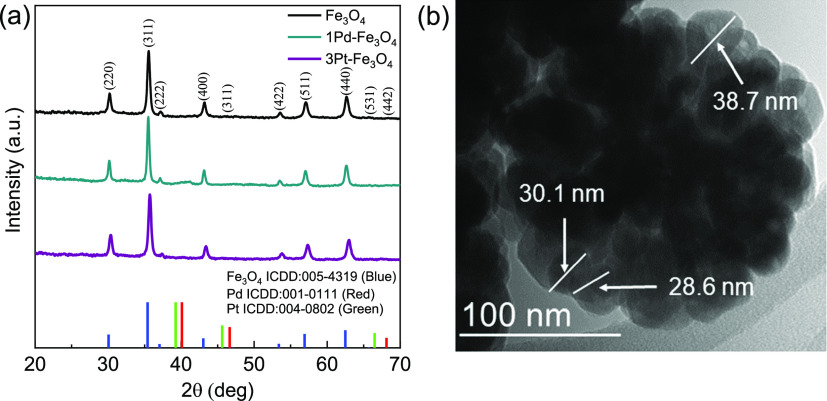
(a) XRD patterns of the Fe_3_O_4_ support, 1Pd–Fe_3_O_4_, and 3Pt–Fe_3_O_4_ where green lines indicate Pt, red Pd, and blue Fe_3_O_4_ peaks and (b) TEM image of the Fe_3_O_4_ support.

The catalysts were characterized via XRD (Figure 2a) to verify the successful synthesis of the Fe_3_O_4_ component. The support diffraction peaks are consistent with the cubic magnetite Fe_3_O_4_ structure (ICDD: 005-4319). An average crystallite size of 21 nm was extracted from the (311) reflection using the Debye–Scherrer equation. In comparison, TEM imaging (Figure 2b) shows spherical nanoparticles between 28 and 39 nm clustered together into larger (100–200 nm) structures. The individual particles are within the ferrimagnetic range (20–128 nm) for hysteresis heating.^27^ There are minimal changes to the diffraction peaks upon addition of the metals, indicating that the Fe_3_O_4_ nanoparticles are stable during the deposition and reduction processes.

**Figure 3:**
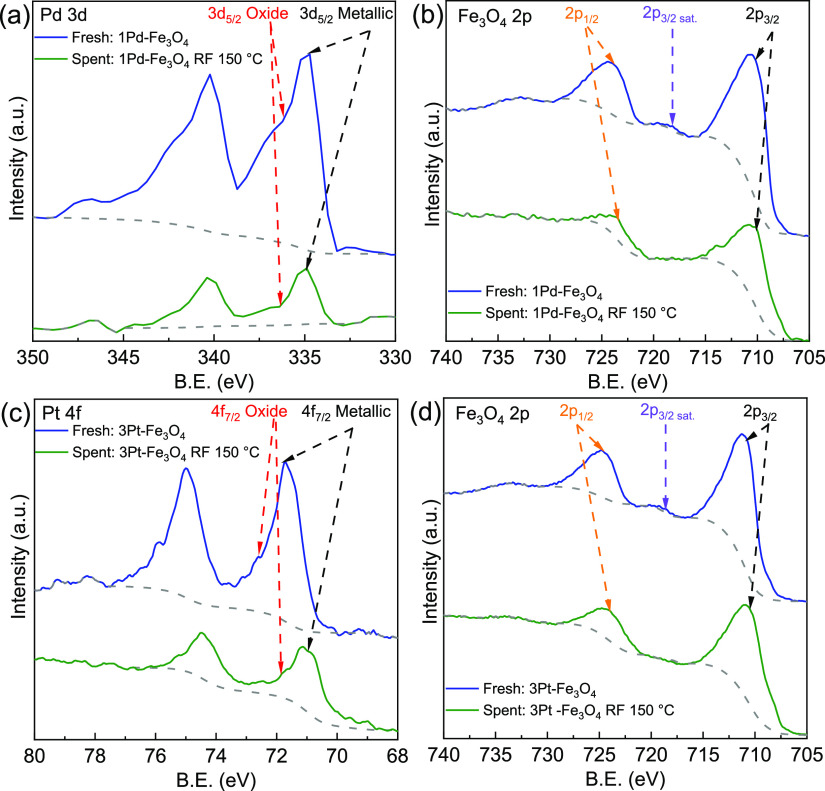
XPS spectra of fresh (blue) and spent (green) catalysts from the RF 150 °C runs: (a) 1Pd–Fe_3_O_4_, Pd 3d, (b) 1Pd–Fe_3_O_4_, Fe 2p, (c) 3Pt–Fe_3_O_4_, Pt 4f, and (d) 3Pt–Fe_3_O_4_, Fe 2p.

The electronic state of fresh and spent (RF, 150 °C) catalysts was probed by XPS. The spectrum of the fresh 1Pd–Fe_3_O_4_ shows a prominent Pd 3d_3/2_ peak at 334.7 eV (Pd^0^) and a small shoulder at 336.2 eV that are characteristic of PdO (Figure 3a).^28,29^ The presence of PdO could arise from both the handling protocols and some intercalation of Pd into the support, with partial electron transfer from Pd to Fe_3_O_4_.^30^ The spent Pd catalyst is electronically similar, with Pd^0^ at 334.9 eV and the PdO shoulder at 336.8 eV (Figure 3a).^28,29,31^ XPS spectrum of the fresh support confirms that it is Fe_3_O_4_ at the surface as the Fe 2p_1/2_ and Fe 2p_3/2_ binding energies are at 723.9 and 710.2 eV, respectively (Figure 3b).^32^ A minor Fe 2p_3/2s_ satellite peak is seen that is characteristic of Fe_2_O_3_.^32^ After use, there is no change in the Fe_3_O_4_ oxidation state. However, the 2p satellite peak is no longer observable, suggesting that Fe_2_O_3_ originally present was reduced to Fe_3_O_4_.

The XPS spectrum of fresh and spent 3Pt–Fe_3_O_4_ showed similar characteristics to that of 1Pd–Fe_3_O_4_. For the fresh catalyst, the major peak is at 71.7 eV with a small shoulder at 72.6 eV (Figure 3c). The 71.7 eV binding energy is in the range for metallic Pt experiencing a strong metal–support interaction (SMSI), while the shoulder is attributed to PtO.^33−35^ The spent Pt is more metallic but continues to demonstrate SMSI with the major peak at 70.8 eV and a minor oxide peak at 71.9 eV (Figure 3c). As seen with the Pd catalyst, the support is almost entirely Fe_3_O_4_ with 2p binding energies of 724.8 and 710.8 eV and a small satellite at 718.6 eV (Figure 3d).^32^ The Fe in the spent catalysts remains as Fe_3_O_4_ (724.0 and 710.5 eV) with a reduction of Fe_2_O_3_ consistent with the disappearance of the satellite peak (Figure 3d), as seen with 1Pd–Fe_3_O_4_. The hydrogenation reactions do not to appear to have significantly affected the electronic states of the two metals.

To quantify the dispersion of the metals and the substrate surface area, chemisorption and physisorption measurements were performed on the pure and decorated substrates. These measurements are shown in Table 1. The Fe_3_O_4_ support showed no measurable CO or H_2_ uptake at ≤100 °C, as expected. The surface area and pore volume increased slightly upon the addition of the Pd clusters. At 50 °C, a Pd dispersion of 19% was measured corresponding to an average (spherical) Pd size of 5.8 nm using
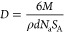
1where *D* is the dispersion (%), *M* is the atomic weight, ρ is the density of the metal, *N*_a_ is Avogadro’s number, *S*_a_ is the area of each surface atom, and *d* is the diameter (nm).^36−38^ The surface area and pore volume of the Pt catalyst both decreased as expected for incipient wetness impregnation. No measurable CO or H_2_ uptake was found on the Pt catalyst at 23, 50, or 100 °C; therefore, dispersion was calculated approximately from the XPS measurement by quantifying the Fe 2p and Pt 4f peaks, relating the peak areas to moles of each element, and dividing the calculated exposed Pt from XPS by the total number of Pt. This lack of adsorption under these conditions has been observed previously and is attributed to Pt-FeO_*x*_ SMSI,^39^ as observed in the XPS spectra. ICP-OES performed on 1Pd–Fe_3_O_4_ and 3Pt–Fe_3_O_4_ gave 1.6 wt % Pd and 3.0 wt % Pt, respectively, suggesting that an additional Pd precursor was deposited, but the target impregnation of Pt was achieved. These values were then used in the calculation of dispersion and average particle diameter.

**Table 1:** Surface Characteristics of Catalysts

catalysts	surface area (m^2^/g)	pore volume (cm^3^/g)	dispersion % (CO)	avg. particle diameter (nm)	ICP: Pd or Pt (wt %)
Fe_3_O_4_	32	0.090	a		
1Pd–Fe_3_O_4_	37	0.096	12	9.2	1.6
3Pt–Fe_3_O_4_	12	0.074	31^b^	3.6	3.0

a No measurable uptake at 23 and 50, 100 °C.

b Dispersion determined by XPS.

To confirm the above XRD results, specifically the relative lack of Fe_2_O_3_, TPO was performed on the uncoated substrates. This compositional analysis is important as the high temperatures required for catalytic reactions require a Fe_3_O_4_/γ-Fe_2_O_3_ (magnetic moment per formula unit: 4.0 μ_β_/2.5 μ_β_) ratio greater than 0.6.^40−43^ A high-temperature N_2_ treatment was used to remove any residual organics while minimizing support oxidation.^25,44^ The TPO was compared to that of commercial Fe_3_O_4_ (Alfa Aesar, 97%, 50 nm) powders. Both were heated to 400 °C with N_2_ to desorb residual hydrocarbons from the syntheses, and then, at 400 °C, the gas was switched to air. By switching to air, the percentage of Fe_3_O_4_ was estimated by utilizing the weight change due to oxidation and the theoretical weight change to Fe_2_O_3_. It was estimated that the synthesized FeO_*x*_ support contains at least 70% Fe_3_O_4_. In contrast, a typical commercial Fe_3_O_4_ powder contained only 57% Fe_3_O_4_. A temperature-field strength calibration was performed by dispersing 50 mg of Fe_3_O_4_ in dodecane. Bulk temperatures were measured by submerging an IR fiber optic probe in the solution. These solvent and catalyst loadings were typical for the hydrogenation of oleic acid to stearic acid, oleyl alcohol, or octadecanol as were the three characteristic temperatures (70, 120, and 150 °C).^45−47^ The solutions rapidly reached the desired temperature (∼5 min) upon exposure to the magnetic fields (Table S1). The solvent could be heated to the desired temperatures with RF fields <20.0 mT under these conditions. The maximum temperature is normally sufficient for more than 80% oleic acid conversion in 4–6 h.^48^ SLP measurements were performed on the Pd and Pt catalysts to characterize the heat generated between the range of 10.8 and 21.2 mT (Figure S2).^44^ At 21.2 mT, 130 ± 13 and 93 ± 9.3 W/g were measured for 1Pd–Fe_3_O_4_ and 3Pt–Fe_3_O_4_, respectively, and are comparable to the previously reported SLP values.

### 3.2 Catalyst Performance

**Figure 4:**
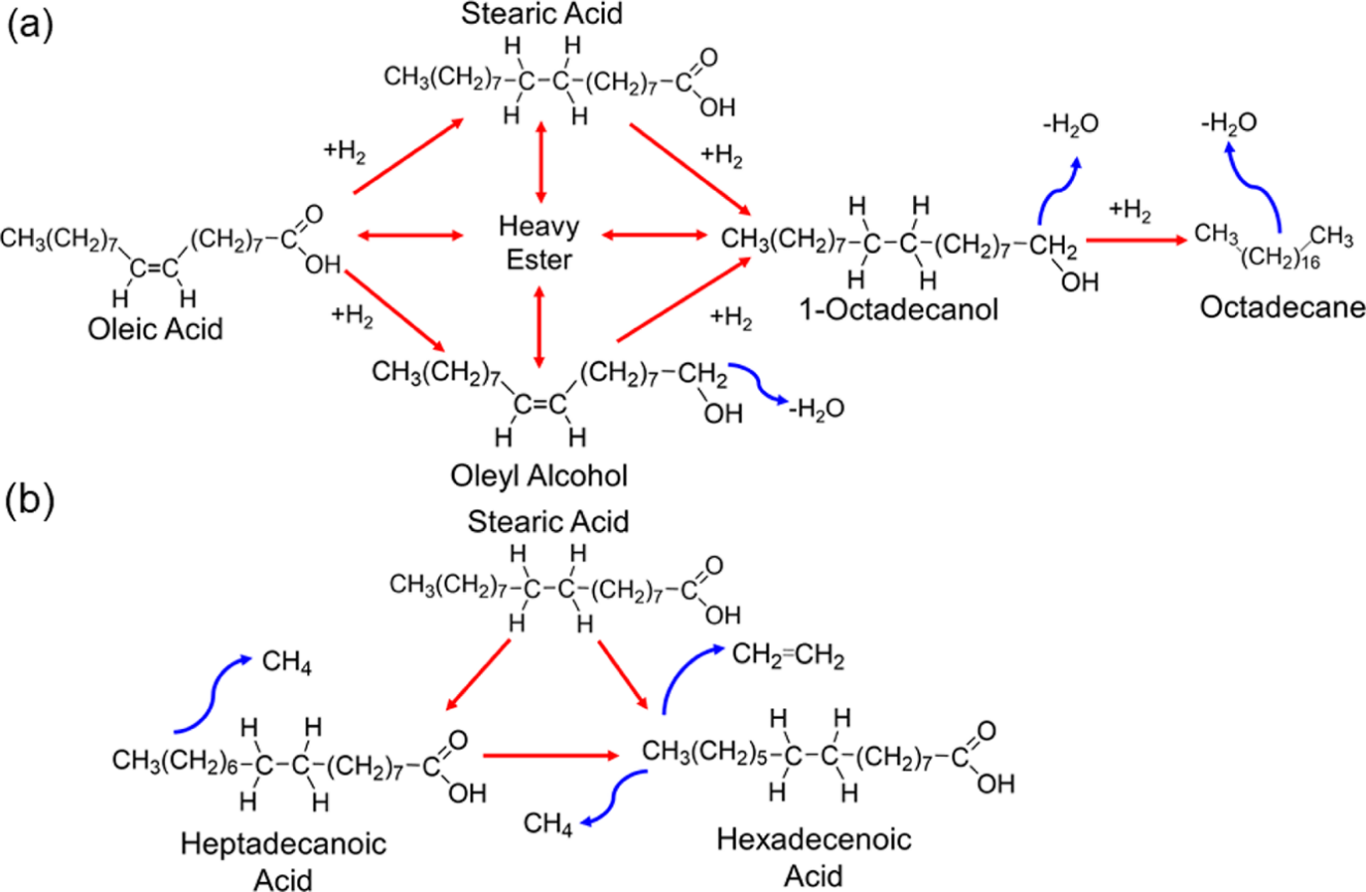
(a) Hydrogenation pathway for oleic acid to its alkane, octadecane,^46^ and (b) cracking pathway for stearic acid to heptadecanoic and hexadecenoic acids.^7,49^

The hydrogenation of oleic acid can produce a saturated acid (stearic acid), an unsaturated alcohol (oleyl alcohol),^46^ a saturated alcohol (octadecanol), and more fully hydrogenated products such as alkanes (Figure 4a). Acidic sites on the catalyst can also result in acid–alcohol condensation to heavy ester byproducts.^46^ The fatty acid can also crack to lighter acids such as heptadecanoic and hexadecenoic acids (Figure 4b).^7,49^ Typical pathways for Pt- and Pd-decorated substrates at high H_2_ pressures are saturated alcohols and saturated acids, respectively. Due to the reactor setup, it was not possible to generate high H_2_ pressures. Instead, H_2_ was bubbled through the solvent solution at slightly above atmospheric pressure. A blank reaction with only the Fe_3_O_4_ support was performed at 170 °C for 4 h (with both conventional and RF heating), with no oleic acid conversion detected for either approach.

**Figure 5:**
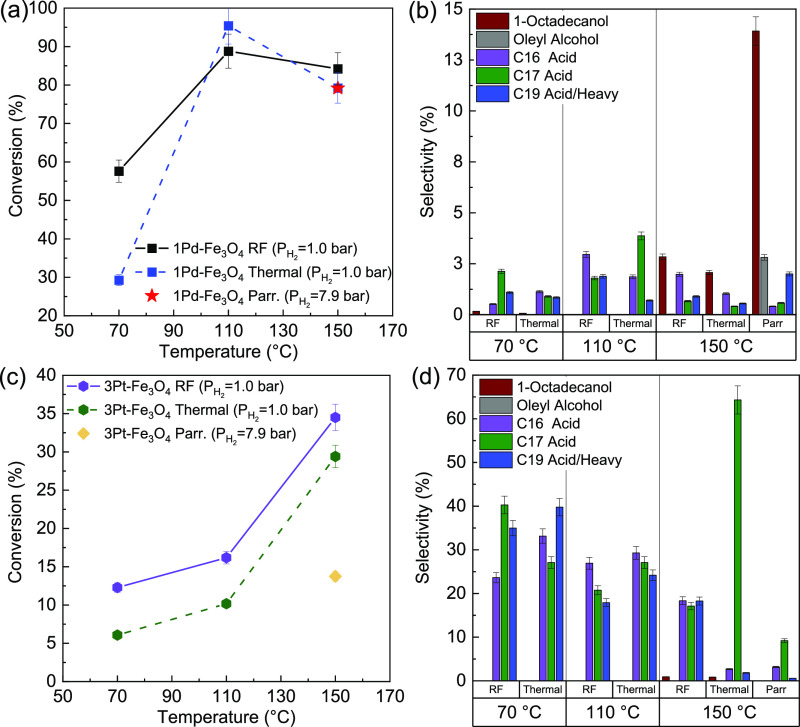
(a) Conversions and (b) product selectivities (balance stearic acid) for 1Pd–Fe_3_O_4_ by different heating methods and (c) conversions and (d) product selectivities (balance stearic acid) for 3Pt–Fe_3_O_4_ by different heating methods. H_2_ pressure = 1.0 bar for RF and thermal energy input. H_2_ pressure = 7.9 bar for thermal reaction in the Parr bomb.

The catalytic results for the 1Pd–Fe_3_O_4_ and 3Pt–Fe_3_O_4_ catalysts are shown in Figure 5. At a 70 °C reaction temperature, the 1Pd-Fe_3_O_4_ catalyst under RF heating converted ∼60% of the oleic acid, 28% higher than that with conventional heating (Figure 5a). However, the conversions converge at higher temperatures (110 and 150 °C), plateauing around ∼85% conversion. The thermodynamic equilibrium conversion of oleic acid calculated using ASPEN-HYSYS is 100% under these conditions. As such, the plateau is attributed to kinetic limitations associated with the reactor. To determine the effects of H_2_ pressure, and thus surface concentration, and compare these catalysts to the literature, a Parr bomb (autoclave) was heated thermally at 7.9 bar H_2_ pressure, 150 °C.^4,50^ The increase in pressure (coverage) showed no effect on conversion but changed the selectivity to 1-octadecanol from 2 to 14% (weight basis, Figure 5b). At atmospheric H_2_ pressure, no effects on product selectivity were observed with the different heating methods. Both were highly selective (>90%) to stearic acid with minimal production of side products (C16, C17, and C19 acids and heavy products of molecular weights 242 and 338 g mol^–1^) at all three bulk temperatures.

It has been previously reported that hydrogen partial pressure can affect the selectivity and activity depending on the metal/support.^50^ For 1Pd–Fe_3_O_4_, the increase of the H_2_ partial pressure increased the product selectivity to the saturated alcohol. Since the catalyst activity did not change, the increased ratio of hydrogen to other adsorbed species on the catalyst must be responsible for the change in selectivity.^51,52^ It is known that monometallic Pd prefers to hydrogenate the C=C bond in aliphatic and aromatic carboxylic acids and aldehydes.^5,10,48,53^ However, Neri et al. have shown that for Pd/Fe_2_O_3_ (even at 1 bar H_2_), strong metal–support interaction can result in electron donation to the metal, favoring the adsorption of the C=O group and increasing selectivity to alcohols.^10,54,55^ Since neither RF nor thermal heating modes showed selectivity to alcohols at 1 bar, there is likely no Pd–Fe interaction here.

Conversely, the 3Pt-Fe_3_O_4_ catalyst was significantly less active and produced more cracked/heavy acids and heavy products than the 1 wt %Pd-Fe_3_O_4_ catalyst (Figure 5c). However, RF heating increased the conversion by 5–6% over conventional heating at each temperature. Performing the hydrogenation with a H_2_ pressure of 7.9 bar at 150 °C in a Parr bomb reduced the activity by a third but with fewer side products (Figure 5d). The higher concentration of hydrogen on the surface decreased the activation energy of hydrogenation while blocking ketonization sites.^39^ Increased H_2_ pressure increases selectivity to stearic acid at the expense of decreasing the catalyst activity.^51,56^

The selectivities of the Pt catalyst were also affected by the heating method. At 70 °C, less than 2% stearic acid was found for both conventional and RF heating, but at 110 °C, the stearic acid selectivity increased to 34% for RF *versus* 19% for conventional heating (a 1.8-fold increase in selectivity). Selectivity to stearic acid increased proportionally with the reactor temperature, ultimately reaching 45% for RF heating *versus* 30% for conventional heating. Therefore, RF was able to decrease the yield of unwanted products and increase selectivity to stearic acid for at least one catalyst.

The literature suggests that Pt/Fe_2_O_3_ catalysts can selectively hydrogenate the C=O bonds of carboxylic acids.^6,39,57,58^ However, this requires a Pt–Fe alloy to be formed during the H_2_ reduction of the catalyst.^39^ Studies have shown that the rate and degree of reduction depend heavily on temperature, reducing gas partial pressure, and concentration of additional elements.^59−61^ The reason why Fe_2_O_3_ can form the Pt–Fe alloy is that when it is reduced (e.g., 450 °C, H_2_ 50 sccm, 1 h), both Fe_3_O_4_ and Fe^0^ are produced.^39^ The Fe^0^ formed then alloys with Pt.^39,57^ The slight shift in binding energies for the used catalyst and the disappearance of the Fe_2_O_3_ satellite peak may be an artifact of this Pt-Fe interaction (Figure 3c,d). In contrast, when starting with Fe_3_O_4_, the reducing conditions (475 °C, 5% H_2_ 30 sccm, 3 h) used here were insufficient to reduce to FeO/Fe^0^. For the reduced Pt/Fe_3_O_4_, no FeO or Fe diffraction peaks were observed.

**Figure 6:**
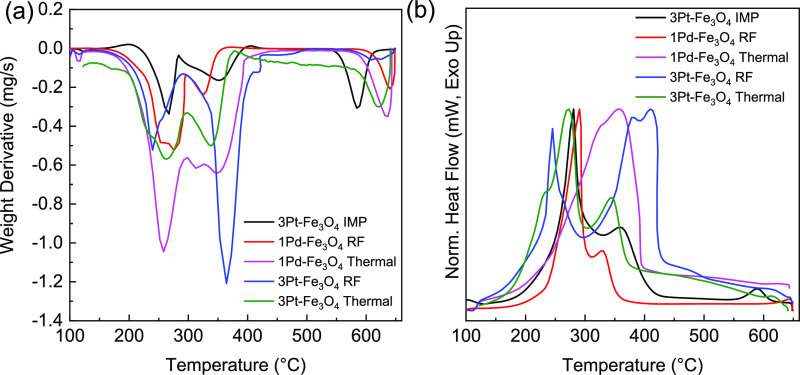
TPO (air, 10 °C/min) for the stearic acid-impregnated fresh 3Pt–Fe_3_O_4_ catalyst and spent RF and thermal catalysts (150 °C): (a) weight derivatives and (b) normalized heat flows (exotherms upward).

TPO measurements were performed to quantify coke deposition on the used (150 °C) 1Pd–Fe_3_O_4_ and 3Pt–Fe_3_O_4_ catalysts. The weight derivatives (Figure 6a) and the normalized heat flow (Figure 6b) of the oxidized catalysts were plotted. The oxidation of the coke/high-molecular-weight carbonaceous species began around 325 °C, which is typical for heavy products from the hydrogenation of aromatic and fatty acids,^62−64^ as confirmed by the TPO of a stearic acid-impregnated 3Pt–Fe_3_O_4_ catalyst. The coke that is oxidized below 550 °C is likely polymeric (C_*n*_H_*m*_) while that oxidized at the higher temperatures is more graphitic.^64,65^ The exotherm seen at 570 °C and above is due to the exothermic decomposition of γ-Fe_2_O_3_ to α-Fe_2_O_3_.^66^ The weight loss and endothermic behavior at these temperatures are due to the reduction of Fe_2_O_3_ to Fe_3_O_4_ and Fe_3_O_4_ to FeO.^67,68^ For RF heating, no coke was found on the spent Pd catalyst, whereas conventional heating resulted in 6.5 wt % of coke relative to the catalyst weight (Table 2). When hydrogenating fatty acids with a Pd catalyst, heavy esters and other heavy products from alkylation, aldol condensation, and Michael addition reactions are known to behave like coke.^7,8,46,69−72^ Additionally, these side products can go through further aromatization and polymerization reactions to form coke.^7,73^ Therefore, the RF heating prevents the formation of heavy esters and other heavy products that would typically form on a Pd catalyst. Alternatively, the coke analyses on the used Pt catalysts showed the presence of coke for both heating methods, with more (on a weight basis) on the RF-heated sample (Table 2). The higher coking rates are attributed to RF reducing the cracking of oleic acid under these conditions. Extended catalyst lifetimes have been seen in other RF-heated systems^74,75^ and have been attributed to better heating control preventing unwanted side reactions.^76^

**Table 2:** TPO Coking Results from the 150 °C Spent Catalysts

catalyst	heating method	wt % of coke on cat.	coke contribution to conversion (%)
1Pd–Fe_3_O_4_	RF	0.0	0.0
	thermal	6.5	0.32
3Pt–Fe_3_O_4_	RF	11	1.8
	thermal	6.3	1.9

Higher catalytic conversions using RF heating have been shown for reactions such as steam reforming and Fischer–Tropsch syntheses, likely due to the nature of localized heating.^21,77^ For similar bulk temperatures, RF has demonstrated that it can improve conversions from 5 to 25%, depending on the catalyst, compared to those of conventional thermal heating. Such improvements were also observed here for both catalysts. The nature of RF heating allows heat to be generated on the surface of the magnetic catalysts instead of being heated by external sources and transporting heat to the catalyst through traditional means.^15,17,18^ What is likely occurring here for the Pt catalyst (and for the Pd catalyst at 70 °C) is that the surface of the catalysts is at a significantly higher temperature than the liquid bulk (∼20–30 °C).^21,77^ Since the reaction is kinetically limited, any temperature gradient from the catalyst surface to the bulk liquid would increase the observed activity. However, this does not explain the positive effects on selectivity (less coke for the Pd catalyst, more stearic acid for the Pt one) we have observed. We offer these results as proof that RF heating can do more than just enhance catalyst activities through enhanced heat transfer. Moving forward, hierarchical core–shell nanostructures could be used to isolate transition metal-supported catalysts from Fe_3_O_4_ while still maintaining the beneficial heating aspects of the RF system to further control the selectivity of these reactions.

## 4 Conclusions

In summary, 1 wt % Pd and 3 wt % Pt catalysts were prepared on Fe_3_O_4_ mesoporous microspheres composed of 28–32 nm spherical nanoparticles. These two magnetically susceptible catalysts were synthesized to study the effect RF induction heating has on the hydrogenation catalysis of a typical fatty acid. This work revealed that induction heating paired with a catalyst that produces heat locally through magnetic hysteresis losses increased both activity and selectivity for the hydrogenation of oleic acid compared to conventional heating methods. For the Pt catalyst, RF provided a minor increase in activity and larger increases in product (stearic acid) yield. At the two higher bulk temperatures, the selectivity increased by a factor of 1.8. For the Pd catalyst, the RF input roughly doubled the stearic acid yield at the lowest bulk temperature, but there were no effects at higher temperatures due to kinetic limitations. The most significant result was that there were no coke-like heavy products on the RF-heated catalyst, in contrast to conventional thermal heating. This result suggests that RF paired with a ferrimagnetic catalyst can greatly limit the rate of coke formation and therefore extend the catalyst lifetime. The ability of RF induction heating to reduce coke formation will be even more beneficial in reactions with traditionally high rates of coking, such as nonoxidative dehydrogenation.

## Supporting Information Available

Time for the batch reactor to reach equilibrium temperature for each RF field strength and SLP measurements for each catalyst (PDF)
