# Supplementary material for: Induction Heating of Magnetically Susceptible Nanoparticles for Enhanced Hydrogenation of Oleic Acid
Source: ACS Appl Nano Mater. 2022 Feb 17;5(3):3676–85. doi: 10.1021/acsanm.1c04351 (PMC8961733; doi:10.1021/acsanm.1c04351)
Supplement: Supplementary file 1 — an1c04351_si_001.pdf [file an1c04351_si_001.pdf]

# Supporting Information

## Induction Heating of Magnetically Susceptible Nanoparticles for Enhanced Hydrogenation of Oleic Acid

**Cameron L. Roman[1], Natalia de Silva Moura[1], Scott Wicker[2], Kerry M. Dooley[1*], and James A. Dorman[1*]**

[1]Cain Department of Chemical Engineering, Louisiana State University, Baton Rouge, Louisiana 70803, United States
[2]Department of Chemistry, Rhodes College, Memphis, Tennessee 38112, United States

Email: dooley@lsu.edu and jamesdorman@lsu.edu

### Time Required to Reach Temperature Equilibrium Relative to Field Strength

Table S1. Time to equilibrate at 70, 110, 150 ˚C bulk temperatures for 50 mg $Fe_3O_4$ in 5.00 g of dodecane for each respective magnetic field strength.

| RF Field Strength (mT) | Temperature (°C) | Time to Equilibrium (sec) |
|---|---|---|
| 12.3 | 70 | 300 |
| 14.3 | 110 | 360 |
| 20.0 | 150 | 300 |

## SLP Measurement for Each Catalyst

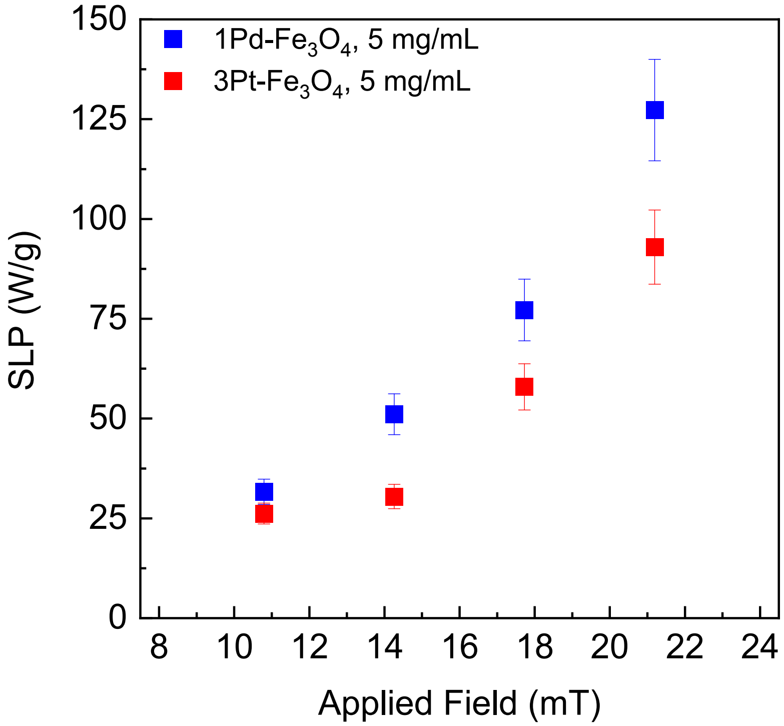


Figure S2. SLP for both 1Pd-$Fe_3O_4$ and 3Pt-$Fe_3O_4$ (5 mg/mL) in water with increasing external applied field.

## Calculation of the catalyst's SLP

SLP was extracted by measuring the time-dependent temperature of the catalyst suspended in water. Measuring the temperature with respect to time, the SLP was obtained from the region with the highest slope before plateauing. The equation used:

$$SLP = \left(\frac{C_{p,Cat}\, m_{Cat} + C_{p,W}\, m_W}{m_{Cat}}\right)\frac{\Delta T}{\Delta t}$$

where $\Delta T/\Delta t$ is the slope of the temperature change with time, and $C_{p,Cat}\, m_{Cat}$ and $C_{p,W}\, m_W$ are the products of heat capacity and mass for the catalyst and water, respectively.
